# Mechanisms involved in cellular ceramide homeostasis

**DOI:** 10.1186/1743-7075-9-71

**Published:** 2012-07-31

**Authors:** M Mahmood Hussain, Weijun Jin, Xian-Cheng Jiang

**Affiliations:** 1 Department of Cell Biology, SUNY Downstate Medical Center, 450 Clarkson Ave, Brooklyn, NY 11203, USA

**Keywords:** Sphingomyelin, Ceramides, Gangliosides, Lipoproteins, Endoplasmic reticulum, Golgi, Plasma membrane

## Abstract

Sphingolipids are ubiquitous and critical components of biological membranes. Their biosynthesis starts with soluble precursors in the endoplasmic reticulum and culminates in the Golgi complex and plasma membrane. Ceramides are important intermediates in the biosynthesis of sphingolipids, such as sphingomyelin, and their overload in the membranes is injurious to cells. The major product of ceramide metabolism is sphingomyelin. We observed that sphingomyelin synthase (SMS) 1 or SMS2 deficiencies significantly decreased plasma and liver sphingomyelin levels. However, SMS2 but not SMS1 deficiency increased plasma ceramides. Surprisingly, SMS1 deficiency significantly increased glucosylceramide and ganglioside GM3, but SMS2 deficiency did not. To explain these unexpected findings about modest to no significant changes in ceramides and increases in other sphingolipids after the ablation of SMS1, we hypothesize that cells have evolved several organelle specific mechanisms to maintain ceramide homeostasis. First, ceramides in the endoplasmic reticulum membranes are controlled by its export to Golgi by protein mediated transfer. Second, in the Golgi, ceramide levels are modulated by their enzymatic conversion to different sphingolipids such as sphingomyelin, and glucosylceramides. Additionally, these sphingolipids can become part of triglyceride-rich apolipoprotein B-containing lipoproteins and be secreted. Third, in the plasma membrane ceramide levels are maintained by ceramide/sphingomyelin cycle, delivery to lysosomes, and efflux to extracellular plasma acceptors. All these pathways might have evolved to ensure steady cellular ceramide levels.

## Introduction

Sphingolipids include hundreds of distinct molecular species that consist of a common eighteen carbon amino-alcohol backbone, sphingosine. Significant information is available about their synthesis and metabolism [1-4]. Sphingolipids, especially ceramides and sphingomyelins, play important roles in maintaining membrane function and integrity. They are found concentrated in lipid rafts, small microdomains in various membranes primarily in plasma membrane that are also enriched in free cholesterol and contain specific proteins [5]. These microdomains are well suited for specialized functions such as cell signaling, lipid and protein sorting, cholesterol efflux and inflammatory response in macrophages and other cell types [5-7].

Ceramides are critical mediators of cellular apoptosis and stress responses [8]. Several mechanisms have been attributed to ceramide-induced apoptosis. Excess amounts of ceramides in the plasma membrane form distinct, cholesterol-poor and ceramide-enriched membrane domains that alter cellular signal transduction by clustering of receptor molecules. These ceramide-rich membrane platforms have been shown to be central in the regulation of apoptosis induced by death receptor activation, stress stimuli and growth factor deprivation as well as in contributing to infection of some pathogens [8]. Further, the contribution of excess ceramides to apoptosis has been reported in several human disorders. Cystic fibrosis transmembrane conductance regulator (CFTR) deficiency results in increases in lung ceramide levels due to alterations in lysosomal pH and is associated with augmentations in cell death, inflammation and infection susceptibility [9]. In Wilson disease, high Cu^2+^ concentrations activate acid sphingomyelinase and increase plasma ceramide levels leading to apoptosis of hepatocyte and erythrocytes [10]. High plasma ceramide levels have been correlated with insulin resistance in type 2 diabetic obese subjects [11] and with increased mortality in sepsis patients [12]. In addition, ceramides mediate inflammatory responses initiated by cytokines or oxidized low density lipoproteins (LDL) [13,14]. Plasma ceramides may contribute to maladaptive inflammation in patients with coronary heart disease [15] and possibly correlate with an increase in LDL oxidation, becoming a risk factor for atherosclerosis [16]. Therefore, ceramides appear to be proatherogenic factors and injurious to arterial walls. Further, there is significant evidence that inhibiting production of ceramides could delay or prevent diseases [17]. In fact, it has been suggested that ceramides are one of the most toxic lipids that can accumulate in the obese [17]. Therefore, their steady state cellular and plasma levels need to be tightly regulated. Here, we briefly summarize what is known about the synthesis and intracellular transport of ceramides and sphingomyelin, present recent observations about changes in tissue and plasma sphingolipid levels in mice deficient in sphingomyelin synthesizing enzymes, and advance a hypothesis that cells have evolved organelle specific mechanisms to control ceramide levels.

## Synthesis and conversion of ceramides into different sphingolipids

### Synthesis of ceramides in the ER and their export

**Figure 1:**
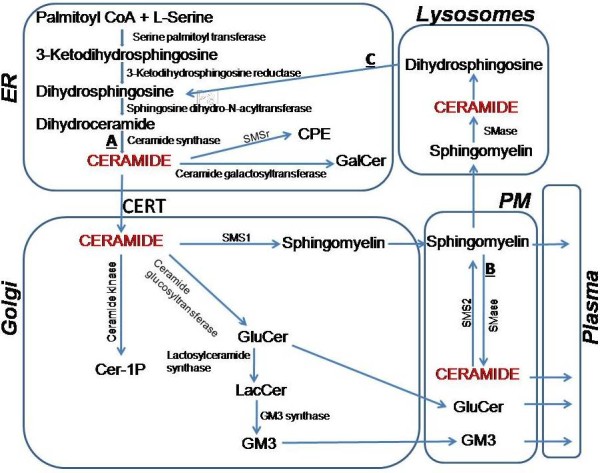
** Pathways involved in cellular homeostasis of ceramides:** The diagram shows the synthesis of ceramides in the endoplasmic reticulum (ER). This involves de novo synthesis **(A)**, hydrolysis of sphingomyelin at the plasma membrane **(B)**, and delivery of sphingolipids to lysosomes **(C)** for recycling. From the ER, ceramides are transported to the Golgi by ceramide transport protein (CERT) and converted to different sphingolipids. These sphingolipids are then transported to the plasma membrane (PM), to plasma, and and to lysosomes. Various mechanisms involved in the transport of ceramides and sphingolipids in different subcellular compartments are discussed in the review. Cer-1P, ceramide 1 phosphate; CPE, ceramide phosphoethanolamine; SMS, sphingomyelin synthase; Galcer, galactosylceramide; Glucer, glucosylceramide; LacCer, lactosylceramide; GM3, ganglioside GM3.

Sphingolipid biosynthesis starts in the endoplasmic reticulum (ER) using non-sphingolipid hydrophilic precursor molecules serine and palmitoyl-CoA [1-3] (Figure 1). Condensation of L-serine and palmitoyl-CoA into 3-ketodihydrosphingosine is facilitated by ER membrane associated serine palmitoyltransferases. Next step in sphingolipid biosynthesis is the reduction of 3-ketodihydrosphingosine to dihydrosphingosine by a reductase. N-acylation of dihydrosphingosine gives rise to dihydroceramide, a product that is still relatively hydrophilic. Conversion of dihydroceramide to ceramides is facilitated by ceramide synthases and involves a desaturation step. Ceramides are hydrophobic and therefore become membrane associated. Apart from this *de novo* synthesis (Figure 1, A), ceramides can also be generated via break down of sphingomyelin in the cell membranes (Figure 1, B) [18] and salvaged from lysosomes (Figure 1, C) after degradation of sphingolipids [17].

Sphingomyelin synthase related protein (SMSr) has no sphingomyelin synthase activity but converts [19,20] some of the ceramide into ceramide phosphatidyletheramine (CPE) whose function is still unknown. Further, ceramides can be converted to galactosylceramides in the brain for myelin synthesis [1,2]. However, the majority of ceramides are transported from the ER to the Golgi by ceramide transport protein (CERT) [21-23].

### Conversion of ceramides to different sphingolipids in the Golgi

In the Golgi, ceramides are converted to sphingomyelin, glucosylceramide and then to more complex sphingolipids such as GM3, or to ceramide-1-phosphate (Figure 1). These products are then transported to plasma membrane, which is the major cellular reservoir for these lipids. The major product of ceramide conversion is sphingomyelin carried out by two sphingomyelin synthase enzymes SMS1 and SMS2 [19,24]. SMS1 is found in the Golgi, while SMS2 is predominantly found in the plasma membranes [19,24]. SMS1 and SMS2 activities are co-expressed in a variety of tissues and cells with different ratios. SMS1 is the major SMS in macrophages [25], while SMS2 is the major enzyme in the liver [26]. Others and we have shown that SMS1 and SMS2 expression positively correlates with sphingomyelin levels in cells and lipid rafts [27-29]. Very little is known about the transport of different sphingolipids out of the Golgi. It is assumed that they are trafficked to plasma membrane via the vesicular transport pathway that carries secretory and plasma membrane proteins.

### Ceramide/sphingomyelin cycle in the plasma membrane

Plasma membrane is enriched in SMS2 that synthesizes sphingomyelin from ceramides as well as in sphingomyelinase (SMase) that hydrolyzes sphingomyelin to ceramides. Thus, plasma membrane ceramide levels are balanced by the activities of these two enzymes. Further, plasma membrane sphingolipids and ceramides can be delivered to lysosomes during the delivery of raft components. It is generally accepted that sphingolipids are hydrolyzed in lysosomes to sphingosine and reutilized for sphingolipid biosynthesis.

### Sphingolipid and ceramide metabolism in the plasma

The major sphingolipid present in the human plasma is sphingomyelin (~90%) and different ceramides constitute the rest of sphingolipids [30]. Sphingomyelin and ceramides are found associated with plasma lipoproteins. The concentrations of these lipids appear to follow the size of the particles; very low density lipoproteins have the highest while high density lipoproteins have the lowest concentrations [30]. Since VLDL concentrations are considerably lower than other lipoproteins, the amounts of sphingolipids carried by these lipoproteins in the plasma are lower than those of LDL and HDL.

There is paucity of knowledge about the metabolism of sphingolipids in the plasma compartment. Due to their structural similarities and localization on the surface of lipoproteins, sphingolipid catabolism is expected to be very similar to phospholipids and free cholesterol. Nascent plasma lipoproteins are hydrolyzed at endothelial cell surfaces by the action of lipoprotein lipases resulting in the hydrolysis of triglycerides and phospholipids and shedding of some of the surface components [31,32]. It is unknown whether sphingolipids remain associated with lipoproteins during and after lipase action. However, it is known that PLTP can transfer sphingomyelin from vesicles to HDL [33]. Hydrolyzed lipoprotein remnants are removed from the plasma via endocytosis involving members of the LDL receptor family [34]. It is likely that some of the sphingolipids are taken by cells during endocytosis of apoB-containing lipoproteins. Thus, sphingolipid catabolism might follow the path of their lipoprotein carriers; however, experimental evidence for this lacking.

## Mechanisms controlling ceramide levels in different subcellular compartments: a hypothesis

**Figure 2:**
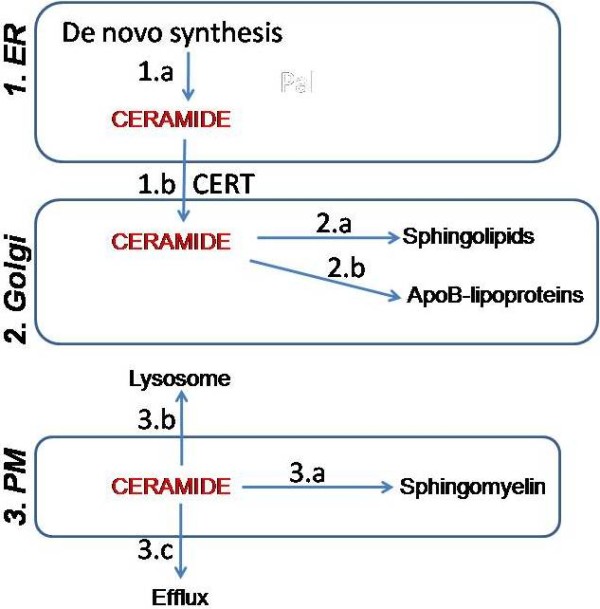
**Hypothesized mechanisms controlling ceramide levels in various subcellular organelles:1.** Control of ceramide levels in the endoplasmic reticulum involves **(a)***de novo* synthesis **(a)** and CERT mediated transfer to Golgi **(b).2.** Regulation of ceramide levels in the Golgi compartment may involve **(a)** conversion to other sphingolipids such as sphingomyelin and glucosylceramides, and **(b)** incorporation into apoB-containing lipoproteins for secretion. **3.** Maintenance of ceramide levels in the plasma membrane (PM) consists of **(a)** conversion to sphingomyelin, **(b)** delivery to lysosomes, and **(c)** efflux to plasma lipoproteins.

To study the importance of sphingomyelin production on cellular and plasma ceramide homeostasis, we generated SMS1 and SMS2 deficient mice with the hypothesis that ablation of these enzymes will lead to excess accumulation of ceramides, cellular apoptosis and embryonic lethality. However, individual knockout of these genes was not embryonic lethal. Analysis of various sphingolipids in macrophages, liver and plasma revealed unexpected knowledge. Individual ablation of these enzymes decreased plasma sphingomyelin by about 50% (Table 1) [25]; therefore, these two enzymes contribute equally to plasma sphingomyelin levels. Surprisingly, SMS1 deficiency had no effect, while SMS2 deficiency increased plasma ceramide levels (Table 1) [25]. More importantly, SMS1, but not SMS2, knockout mice had 4–7 fold increase in glucosylceramide and GM3 in the plasma and tissues. To explain these unexpected observations, we advance the hypothesis that cells have evolved several mechanisms to control ceramide levels in different sub-cellular compartments (Figure 2). First, cells maintain ceramide levels in the ER involving *de novo* synthesis (Figure 2.1a) and protein mediated export (Figure 2.1b). Second, enzymatic conversion of ceramides to various sphingolipids is the major mechanism regulating ceramide levels in the Golgi complex (Figure 2.2a). Further, they can be secreted with apoB-containing lipoproteins in some cells (Figure 2.2b). Third, sphingomyelin/ceramide cycle, lysosomal delivery, and efflux mechanisms might be involved in the control of ceramide levels in the plasma membrane (Figure 2.3).

**Table 1:** Levels of various sphingolipids in wildtype and SMS knockout mice

	**SM**	**PC**	**Cer**	**GluCer**	**GM3**
**Plasma**	nmol/ml			ng/ml	
Wildtype	105 ± 5	1443 ± 109	805 ± 33	3714 ± 358	342 ± 22
*Sms1* KO	52 ± 3*	1322 ± 99	888 ± 80	25705 ± 2317*	1996 ± 219*
Wildtype	95 ± 3	1301 ± 78	796 ± 55	3209 ± 277	329 ± 34
*Sms2* KO	47 ± 3*	1232 ± 102	1032 ± 50*	3638 ± 421	367 ± 51
**Liver**	nmol/mg protein			ng/mg protein	
Wildtype	11 ± 2	102 ± 38	247 ± 29	104 ± 10	22 ± 2
*Sms1* KO	7 ± 1*	108 ± 21	184 ± 21*	396 ± 31*	120 ± 13*
Wildtype					
*Sms2* KO	8 ± 1*	130 ± 15	284 + 42	109 ± 19	19 ± 5
**Macrophages**					
Wildtype	63 ± 7	117 ± 22	985 ± 98	85 ± 17	151 ± 50
*Sms1* KO	19 ± 5*	131 ± 19	1027 ± 128	1019 ± 26*	1255 ± 77*
Wildtype	61 ± 6	157 ± 33	890 ± 109	111 ± 20	103 ± 12
*Sms2* KO	51 ± 2*	139 ± 23	962 ± 91	130 ± 18	99 ± 7

Different sphingolipids were quantified by LC/MS/MS from tissues obtained from wildtype and SMS1 and SMS2 knockout mice. Statistical significance was evaluated using Student *t*-test. Values are mean ± SD, n = 6. *P<0.01. *SM*, sphingomyelin; *PC*, phosphatidylcholine; *Cer*, ceramide; *DHCer*, dihydroceramide; *Glucer*, glucosylceramide; *Sph*, sphingosine; *S1P*, sphingosine-1-phosphate; *Sa1P*, sphinganine-1-phosphate; *GM3*, ganglioside GM3. Data are modified from [25].

### Controlling ceramides in the endoplasmic reticulum

Three different pathways can be envisioned for the transport of ceramides from the ER to Golgi; vesicular transport, incorporation into lipoproteins, and protein mediated transport. Ceramides can be transported to the Golgi as part of the vesicular trafficking that is well described for protein secretion. In this pathway, transport vesicles bud off from the ER and then fuse with *cis*-Golgi. Lipoproteins are assembled in the ER and transported to Golgi via special transport vesicles [35,36]. Obviously, ceramides could become part of lipoprotein and protein transport vesicles and be transported to the Golgi. However, this vesicular trafficking pathway might be slow and not sufficing to control ceramide levels that are actively synthesized in the ER compartment.

A significant understanding about the regulated process of ceramide movement from the ER to Golgi came with the identification of ceramide transfer protein (CERT) that picks up ceramides from the ER membrane and deposits them into the Golgi membrane independent of the vesicular trafficking. CERT is essential for embryonic survival perhaps because CERT deficiency-mediated ceramide accumulation can cause severe developmental disruption without inducing apoptosis [21-23]. These observations highlight the importance of CERT pathway and indicate that vesicular trafficking either as part of membranes or lipoproteins is perhaps not a major pathway for ceramide transport from the ER to Golgi or plasma.

### Regulating ceramides in the Golgi

We envision at least two different mechanisms involved in the control of ceramides in this compartment. First involves vesicular trafficking that is well described for the transport of proteins from the Golgi to the plasma membrane. This process involves exit of vesicles from the *trans*-Golgi and their subsequent fusion with the plasma membrane. Second, at least in lipoprotein synthesizing tissues such as liver, intestine and heart, sphingolipids and ceramides could become part of newly synthesized triglyceride-rich apolipoprotein B-containing lipoproteins in the Golgi. There is significant evidence in the literature for this mechanism. It has been reported that the major carriers of ceramide in the plasma are very low density lipoproteins (VLDL) [30]. Studies in hamsters suggest that *de novo* ceramides are secreted via VLDL/LDL pathway by the liver [37,38]. Moreover, isolated rat hepatocytes have been shown to secrete ceramides as part of apoB-containing particles [39]. Activation of serine palmitoyl transferase, the rate-limiting enzyme in the *de novo* ceramide synthesis by palmitic acid but not other fatty acids elevates VLDL and LDL ceramides. Inhibition of *de novo* ceramide synthesis by Fumonisin B_1_ prevents the incorporation of ceramide in apoB-containing particles [39]. Despite the evidence that hepatocytes secrete ceramides with VLDL, it remains to be determined how ceramides and possibly sphingolipids are deposited in nascent lipoproteins. There are two candidate proteins that could play a role in this process. First, microsomal triglyceride transfer protein [40,41], which transfers several lipids, can transfer sphingolipids and deposit them in nascent lipoproteins. Second, phospholipid transfer protein [33,42,43] can transfer these lipids onto nascent lipoproteins. Alternatively, there might be other yet unidentified proteins that could specifically transfer sphingolipids to lipoproteins. Thus, ceramide levels can be regulated by vesicular trafficking of membranes and lipoproteins.

Due to the possible existence of these two pathways, we had anticipated that SMS1 and SMS2 deficiencies will decrease sphingomyelin and increase ceramides in cells and plasma. To test this, we measured different lipids in the plasma, liver and macrophages of SMS1 and SMS2 deficient mice (Table 1) [25,26]. SMS1 and SMS2 deficiency had no effect on phosphatidylcholine levels. However, their deficiencies had significant effects on cellular and plasma sphingomyelin, ceramide, glucosylceramides and gangliosides such as GM3. The SMS1 deficient mice had normal levels of plasma ceramides. By contrast, SMS2 knockout mice had significantly increased plasma ceramides [25,26]. Since, SMS1 is mainly present in the Golgi, we had anticipated that ceramides would accumulate in cells. Instead, we found significant reductions (−26%, p<0.01) in hepatic ceramides in SMS1 deficient mice. To our surprise, we found that both glucosylceramide and GM3 were dramatically increased (7-fold and 6-fold, respectively) in SMS1 deficient mouse plasma, but not in SMS2 ablated animals (Table 1). Further, glucosylceramide and GM3 were also increased by 4- and 6-fold, respectively, in the livers of SMS1 deficient mice compared to controls due to increases in ceramide glucosyltransferase [25]. Similar changes were also noted in macrophages. However, SMS2 deficiency had no effect on glucosylceramide and GM3 levels in the plasma, liver and macrophages (Table 1) [25]. These data show that SMS1 deficiency increases cellular and plasma glucosylceramide and GM3 with no significant effect on plasma ceramide.

To explain these observations, we propose that Golgi ceramide levels are controlled by their enzymatic conversion to different sphingolipids. Cells have evolved different enzymes to convert ceramides into various sphingolipids (Figure 1). Hence, deficiency in the Golgi sphingomyelin synthesis, as in the absence of SMS1, results in the shunting of ceramides to glucosylceramide and ganglioside synthesis. Based on this hypothesis, we predict that deficiency in glucosylceramide synthesis will increase cellular and plasma sphingomyelin levels. A consequence of enhanced synthesis of different sphingolipids is their accumulation in cells. Obviously, this can be tolerated to some extent and could explain their higher cellular levels. However, we anticipate that excess amounts would be deleterious. Therefore, other mechanisms must exist to lower sphingolipids in the Golgi. This might involve their secretion possibly as part of apoB-lipoproteins and could explain increases in plasma levels of glucosylceramide and GM3 in SMS1 deficient mice. Thus, conversion of ceramides to different sphingolipids and secretion of these sphingolipids with apoB-lipoproteins might be the two major pathways that control Golgi ceramide levels.

### Maintaining plasma membrane ceramide levels

As discussed above, ceramides are likely not brought to the plasma membrane from the ER involving transport vesicles. Instead, plasma membrane ceramides are probably generated from sphingomyelin by sphingomyelinases. It has been known for some time that SMS2 primarily resides in the plasma membrane [19]. Therefore, ablation of SMS2 is expected to increase cellular ceramides due to their increase in plasma membrane. Alternatively, cells might have high glucosylceramides and GM3 as seen in SMS1 KO mice. But, we neither saw significant increases in cellular ceramides nor in glucosylceramide/GM3. No change in glucosylceramide/GM3 could be a consequence of the absence of the enzymes that convert ceramides to these lipids in the plasma membrane. It is possible that plasma membrane ceramides can be maintained by their delivery to lysosomes as part of the endocytic vesicles that bring extracellular or membrane components to lysosomes for destruction or reutilization. In this pathway, sphingolipids are hydrolyzed in the lysosomal compartment and ceramides are salvaged for further utilization. However, this is also expected to increase cellular ceramide levels or sphingomyelin levels due to the presence of SMS1 activity in the Golgi of SMS2 knockout mice. But, we did not observe significant increases in cellular sphingomyelin or ceramide levels. Therefore, there must be other mechanisms that control ceramide levels in the plasma membrane besides their conversion to sphingomyelin and reutilization via lysosomes. To explain this, we propose a third mechanism involving their efflux to plasma lipoproteins. In this process, ceramides can be directly given off from the plasma membrane to extracellular lipoproteins. ATP cassette transport proteins or yet unknown proteins might participate in this process. It has been reported that ABCG1 but not ABCA1 can directly mediate sphingomyelin efflux from cells to extracellular HDL [44]. However, no evidence exists for the efflux of ceramides and deserves further investigation.

## Perspectives

The purpose of this short review is to advance the concept that cells have evolved different mechanisms to control ceramide levels in various subcellular organelles. In the ER, ceramide levels are controlled by its export to the Golgi by CERT. In the Golgi, ceramide levels are controlled by their conversion to sphingomyelin, glucosylceramides and possibly other sphingolipids. In the plasma membrane, ceramide are controlled by their conversion to sphingomyelin and efflux to plasma lipoproteins. Most of the mechanisms proposed in the control of cellular ceramides and sphingolipids can be easily tested. The role of conversion of ceramides to different sphingolipids can be evaluated by ablating critical enzymes involved in sphingolipid synthesis such as we have done for SMS1 and SMS2. We anticipate that ablation of glucosylceramide synthase might increase sphingomyelin levels. The role of apoB-lipoproteins in the secretion of ceramides and sphingolipids can be examined in mouse models that are deficient in apoB, MTP, and/or PLTP. Mechanisms involving cellular efflux of ceramides and sphingolipids can be addressed using mouse models of ABCA1, ABCG1 deficiency. We hope that this review will generate enthusiasm to focus on various sub-cellular mechanisms involved in the regulation of ceramides and other sphingolipids.

## Abbreviations

ER, Endoplasmic reticulum; SMS, Sphingomyelin synthase; LDL, Low density lipoproteins; CERT, Ceramide transfer protein.

## Competing interests

The author(s) declare that they have no competing interests'.

## Authors’ contributions

MMH wrote, edited, and submitted the manuscript; WJ and XJ provided critical comments; the table is from XJ; MMH made the figures. All authors read and approved the final manuscript.
